# Case Report: Clinical case analysis of gaucher disease management in a resource-limited setting: a single center experience from Kashigar, Xinjiang Uygur Autonomous Region, the Western China

**DOI:** 10.3389/fped.2025.1530177

**Published:** 2025-09-22

**Authors:** Gulinuer Maimaititusvn, Maierhaba Kulaixi, Abudouwaili Atawula, Fang Liu

**Affiliations:** ^1^ Department of Pediatrics, Kashi Prefecture Second People’s Hospital, Kashgar, Xinjiang, China; ^2^ Department of Pediatrics, Shanghai East Hospital, Tongji University, Shanghai, China; ^3^ The People's Hospital of Yecheng District, Kashgar Prefecture, Xinjiang Uygur Autonomous Region, China

**Keywords:** gaucher disease, case report, splenomegaly, thrombocytopenia, epistaxis

## Abstract

**Objective:**

This report presents the inaugural case of Gaucher disease identified in Kashgar Prefecture, Xinjiang, the westernmost region of China. It emphasizes an analysis of the clinical characteristics, diagnostic challenges, and treatment strategies within the unique geographical, cultural, and ethnic contexts. The study aims to investigate potential associations between environmental factors, genetic backgrounds, and lifestyle in Kashgar Prefecture, as they relate to the diagnosis, treatment, and prognosis of Gaucher disease, with the goal of optimizing diagnostic and therapeutic approaches in similar regions.

**Methods:**

We performed a retrospective analysis of the patient's clinical data, employing advanced diagnostic methods in conjunction with multidisciplinary collaboration. The data encompassed clinical symptoms, laboratory tests, imaging examinations, genetic testing, diagnostic procedures, and individualized treatment plans.

**Results:**

A 12-year-old female patient from Kashgar, Xinjiang, China, presented with chronic anemia, hepatosplenomegaly, thrombocytopenia, recurrentepistaxis, and osseous pain. She was diagnosed with Gaucher disease type I through genetic testing and enzymatic examination. This case represents the first reported instance of this condition in the Xinjiang region. Notably, it exhibited unique clinical features, including the age of onset, severity of symptoms, and potential regional complications. Treatment with high-dose ambroxol and imiglucerase significantly alleviated the patient's symptoms, and continuous follow-up was conducted to assess long-term efficacy.

**Conclusion:**

This report underscores the critical importance of early diagnosis and timely intervention in the management of Gaucher disease, particularly in regions with limited medical resources such as Kashgar. The successful diagnosis and treatment of this case have facilitated communication and cooperation between primary healthcare units and external medical institutions. This has further driven interactions in various aspects such as academic exchanges, teleconsultations, and medical assistance. In turn, this has provided a solid foundation for safeguarding patients’ rights and improving medical services.

## 1 Introduction

### 1.1 Main medical history and important auxiliary examinations

On November, 2023, a 12-year-and-4-month-old girl was admitted to the pediatric endocrinology and metabolism ward of Xinhua Hospital, affiliated with Shanghai Jiaotong University, due to five-year persistent hepatosplenomegaly that had worsened in the past year. Since the age of 3–4, she had intermittent epistaxis. Five years ago, during an examination at the Second People's Hospital of Kashi District for epistaxis and low back pain, hepatosplenomegaly and anemia were diagnosed.

**Figure 1:**
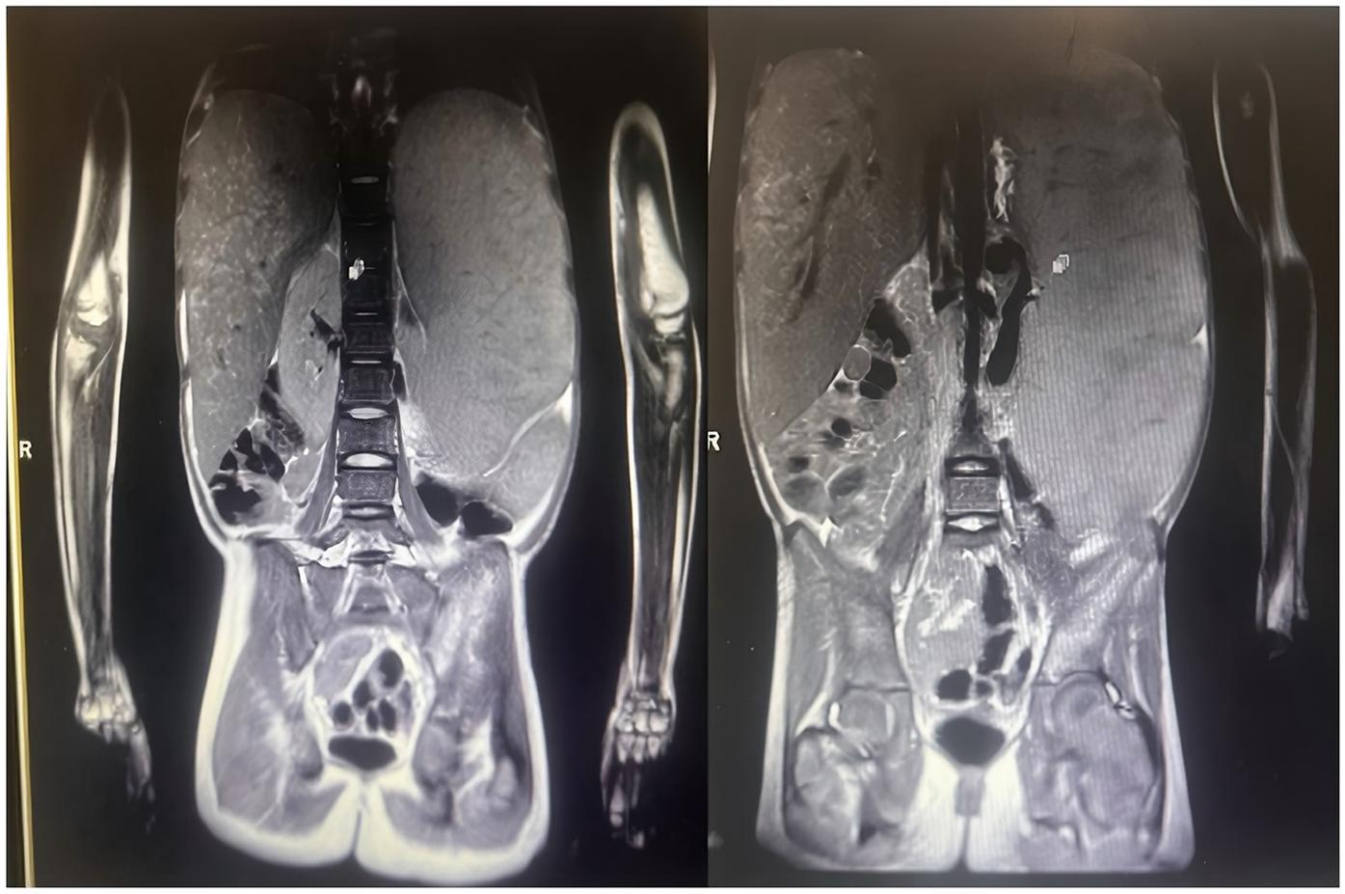
Abdominal MRI (2024-01-19) **Liver**: enlarged liver volume, with no significant widening of the liver fissures. No apparent dilation of the intrahepatic bile ducts. The liver's right lobe shows heterogeneous signals, with patchy areas exhibiting slightly high signals on T2-weighted imaging (T2WI). No significant abnormalities detected in the bile ducts. **Spleen**: Enlarged spleen, with homogeneous signals in the parenchyma. The splenic hilum shows dilation of the veins, with the widest part measuring approximately 12 mm. **Kidneys**: Both kidneys exhibit normal morphology and signals, with no significant abnormalities. **Left Hip Bone**: High signal on T2WI, indicating possible pathology such as edema or inflammation.

Bone marrow puncture showed Gaucher cells, and genetic testing identified GBA gene variations (E7 missense variation c.907C > A and E9 nonsense variation c.1259G > A), confirming Gaucher disease. Due to financial issues, she didn't receive regular treatment. In the past year, her liver and spleen enlarged progressively, with the liver 6.5 cm below the costal margin and the spleen about 26 cm into the pelvic cavity. The specific dimensions are as shown in the abdominal MRI results (Figure 1). She also had abdominal distension, intermittent epistaxis, anemia (Hb 86 g/L), bone pain, growth retardation (height 134 cm, 3.16 SD below the mean; bone age 8–9 years), and delayed puberty (Tanner breast stage B1). GBA gene sequencing detected a heterozygous missense variant [c.907C > A, p.(Leu303Ile)] on exon 7 from her mother and a heterozygous nonsense variant [c.1259G > A, p.(Trp420*)] on exon 9 of unknown origin. Leukocyte—based glucocerebrosidase activity was 2.16 nmol/L (reference range: 8; normal range: 6.56–55.1 nmol/L), far below normal, strongly indicating Gaucher disease.

### 1.2 Analysis of disease progression delay

Since the patient was diagnosed with Type I Gaucher disease at a local hospital in September 2019, enzyme replacement therapy (ERT) could not be initiated due to social and economic constraints. The patient has long relied on palliative supportive care provided by primary medical institutions, which included intermittent blood transfusions to correct anemia, pain management, and infection prevention and control. The disease progression period began in October 2023. Through the intervention of a multi-level medical assistance system—including special support from the Chinese Rare Disease Network, coverage by the urban and rural residents' serious illness medical insurance policy, and targeted grants from local charitable foundations—cross-regional referral to the Rare Disease Center of Shanghai Children's Medical Center was finally achieved. Within the framework of a standardized multidisciplinary team (MDT), the patient received a comprehensive management plan based on evidence-based medicine, which included the adjuvant therapy of mucolytic ambroxol (initial dose 5 mg/kg·d) to improve lysosomal function, combined with iron, calcium, and vitamin D3 metabolic support. Simultaneously, the evaluation and financing plan for disease-modifying therapy was initiated. This case underscores the vital role of a multi-layered rare disease security framework—linking government, charitable organizations, and medical insurance—in eliminating barriers to treatment accessibility. Moreover, it offers a practical policy example for implementing targeted medical interventions that benefit socially and economically vulnerable groups.

### 1.3 Treatment process and efficacy evaluation

**Figure 2:**
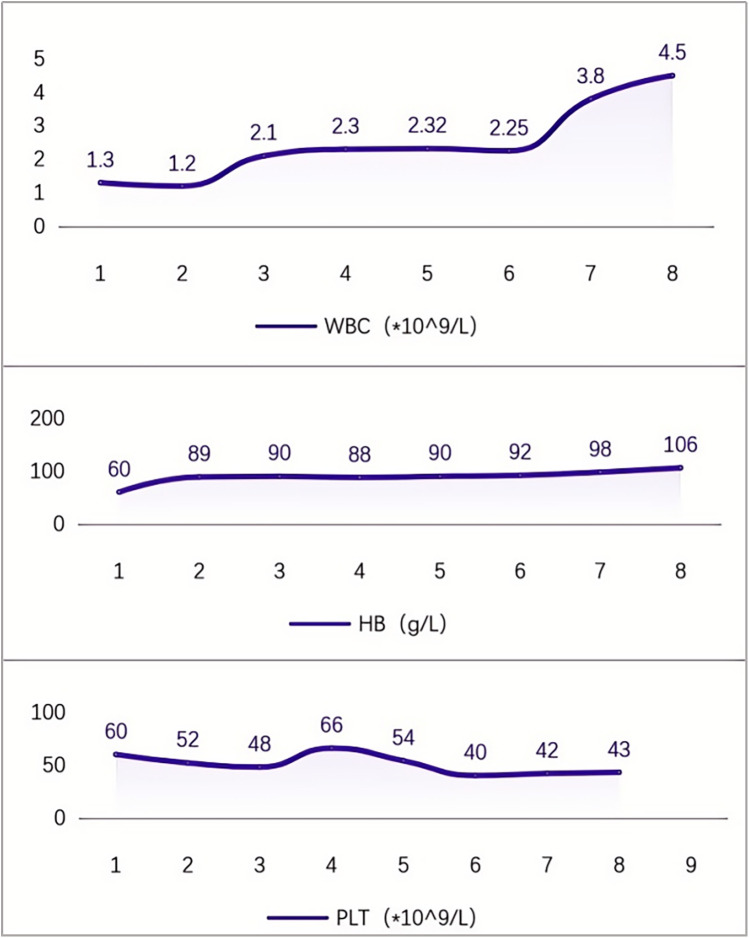
Changes in blood cells during 6 months of medication for Gaucher disease (remarks: (1). At discharge; (2). 1 week after medication initiation; (3). 1 month after medication initiation; (4). 2 months after medication initiation; (5). 3 months after medication initiation; (6). 4 months after medication initiation; (7). 5 months after medication initiation; (8). 6 months after medication initiation).

**Figure 3:**
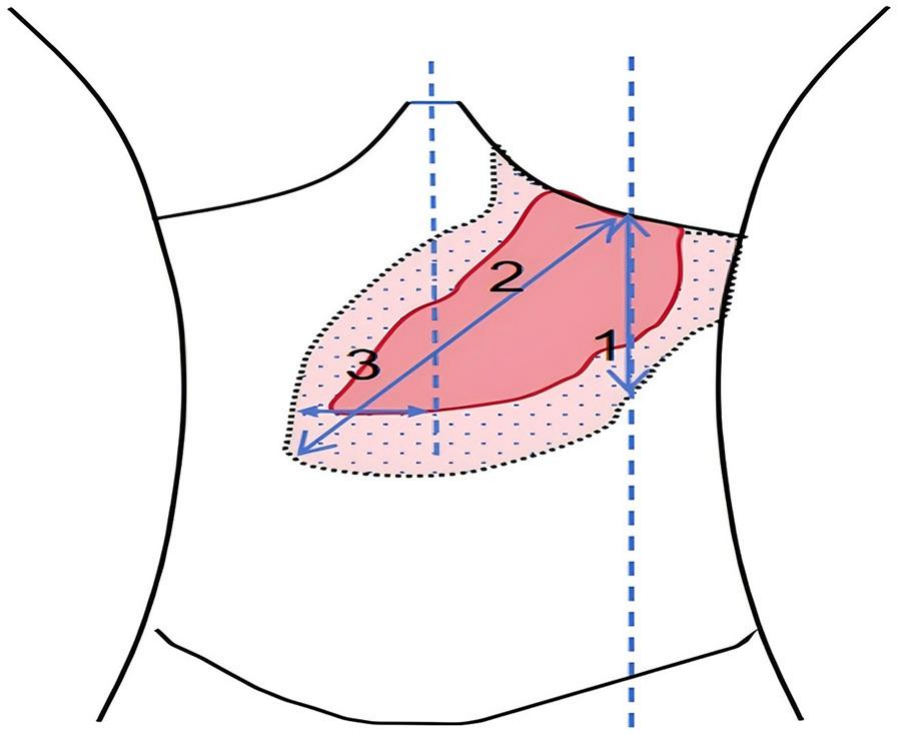
After treatment with high-dose ambroxol, the changes in spleen measurement values were as follows: in group I, the measurement decreased from 23 cm to 22 cm, with *P* > 0.05; in group II, it decreased from 26 cm to 23 cm, with *P* = 0.03; and in group III, it decreased from 8.5 cm to 7.0 cm, with *P* = 0.02. Additionally, the abdominal circumference decreased from 67 cm before treatment to 64 cm. The liver volume also decreased by 21.1%, with the measurement dropping from 6.5 cm below the right costal margin to 5.0 cm and from 12 cm below the xiphoid process to 10 cm, with *P* = 0.01.

After being discharged from the hospital, the pediatric patient received ambroxol in combination with supportive treatment for six months, resulting in significant improvements across multiple systemic indicators. All effectiveness indicators were systematically organized, and the changes in data before and after treatment were quantified using the effective increase rate of clinical indicators (for details, see Table 1 and Figure 2). The results of hematological examinations indicated that the white blood cell count increased from a baseline value of 1.30 × 10^9^/L to 4.50 × 10^9^/L, and the hemoglobin concentration rose from 60 g/L to 106 g/L, suggesting effective alleviation of bone marrow suppression and hemolytic conditions. However, the fluctuating changes in platelet count indicated that hypersplenism associated with massive splenomegaly persisted. Imaging evaluations demonstrated significant decreases in the volumes of the spleen and liver, as well as in abdominal circumference, confirming that the intervention on lysosomal function had a reversing effect on visceral infiltration. Regarding clinical symptoms, the frequency of epistaxis significantly decreased, the volume of bleeding was reduced, and bleeding episodes could resolve spontaneously. Furthermore, bone pain and limitations in movement were also markedly alleviated (for details, see Table 1 and Figure 3).

**Table 1:** Changes in Various analytical indicators at diagnosis and during follow-up and variables of imaging follow-up.

Category of analysis indicators	Baseline (at diagnosis)	1-month follow-up	3-month follow-up	6-month follow-up	Improvement status
Hematological Indicators					
WBC (*109/L)	1.3	2.1	2.32	4.5	WBC increased effectively by 2.46 times
HB (g/L)	60	90	90	106	HB increased by 76.67%
PLT (*109/L)	43	48	54	43	PLT showed fluctuating changes
Imaging Indicators					
Long Diameter of Spleen (cm)	26			23	Spleen volume decreased by 30.30%
Thickness of Spleen (cm)	8.5			7	
Subcostal Liver (cm)	6.5			5	Liver volume decreased by 21.1%
Subxiphoid Liver (cm)	12			10	
Abdominal Circumference (cm)	67			64	Abdominal circumference decreased
Clinical Symptom Indicators					
Epistaxis Frequency	2–3 times/2–3 days	1 time/7–10 days	1 time/1–2 weeks	1 time/1–2 weeks	Symptoms of epistaxis and bone pain relieved; no obvious limitation of movement
Single Bleeding Volume	15 ml	10 ml;	<10 ml;	≤6 ml;	
Bone Pain	Significant bone pain	Significant bone pain	Intermittent bone pain	basically relieved	
Degree of Movement Limitation	Yes	Yes	No	No	

Based on the stage-based efficacy, since July 2024, the patient has started receiving enzyme replacement therapy with imiglucerase (1,200 units per administration) to regulate the level of glucosylsphingosine and delay the progression of the disease. The current treatment relies on the collaborative follow-up system between the county hospital and the regional rare disease center. By continuously monitoring the routine blood tests, liver and spleen ultrasound, and metabolic indicators, the management of treatment compliance is strengthened.

Overall, in this case of type I Gaucher disease, after 6 months of ambroxol treatment, hemoglobin and white blood cell levels showed significant improvement (*P* < 0.001), clinical symptoms were alleviated, but the reduction in spleen volume was limited (26 cm→23 cm), meeting the criteria for SRT/ERT upgrade. In July 2024, imiglucerase combination therapy was initiated. In subsequent treatment follow-up, it is necessary to dynamically monitor immune antibodies and Lyso-GL1 levels to optimize the dose and prevent secondary resistance.

## 2 Diagnosis and genetic mechanism analysis of GD

### 2.1 Epidemiology and pathological mechanisms of gaucher disease

GD is an autosomal recessive lysosomal storage disorder triggered by mutations in the GBA gene (OMIM 606463), which leads to a deficiency in the activity of β-glucocerebrosidase (GCase) (1, 2). The dysfunction of GCase promotes the accumulation of glucocerebroside within the lysosomes of mononuclear-macrophage cells, giving rise to the formation of “Gaucher cells”. Through mechanisms such as cytotoxicity, inflammatory responses, and tissue infiltration, this process can lead to manifestations including hepatosplenomegaly, hematological abnormalities, bone diseases, and neurodegeneration (3–5). There are significant ethnic disparities in the incidence of GD. The prevalence rate is as high as 118 per 100,000 individuals in the Eastern European Jewish population. In contrast, in the eastern China region and Taiwan area of China, the prevalence rates are 1 in 80,855 and 1 in 10,313, respectively (6, 7).

### 2.2 Clinical classification and genetic characteristics

GD is classified into three types according to the degree of nervous system involvement. Type I (non-neuropathic type), accounting for more than 90%, is characterized by hepatosplenomegaly, cytopenia, and bone diseases. Type II (acute neuropathic type) is marked by rapidly progressive brain injury, while Type III (chronic neuropathic type) is manifested as delayed neurodegeneration (8). Mutations in the GBA gene, including missense, nonsense, and frameshift mutations, lead to the accumulation of toxic metabolites such as glucosylsphingosine (Lyso-GL1) (5). To date, more than 400 GBA mutations have been identified. Among the Chinese population, there are approximately 40 types of mutations. The L444P mutation is a common mutation that causes neurological symptoms (9). In pediatric patients, S (C.1226A > G), L444P (C.1448T > C), IVS2 + 1 (C.11511G4A), and 84GG (C.84dupG) are high-frequency allelic variants (10). Pathogenic variants of GBA1, as well as mutations such as F76V, N227S, R87W, M75V, and R392W, are associated with milder symptoms (11). On the other hand, mutations like L483P, RecNcil, and R535C are related to severe phenotypes (12–14).

### 2.3 Diagnostic strategies and research prospects

The diagnosis of Gaucher disease (GD) primarily involves enzyme activity detection [characterized by β-glucocerebrosidase (GCase) activity below 15% of the normal value] and gene sequencing. Notably, in cases where GD is suspected, it is advisable to conduct enzymatic evaluations for both GD and Niemann-Pick disease (NPD), given the overlapping clinical and biochemical features between these two lysosomal storage disorders (7, 15, 16). Enzyme activity detection serves as the gold standard, while gene sequencing aids in genetic counseling and prognosis assessment. Plasma glucosylsphingosine (Lyso-GL1), due to its high sensitivity and specificity, has been incorporated into the diagnostic and therapeutic monitoring algorithms for GD (16–20). In contrast, chitotriosidase, although a potential biomarker, is limited by genetic polymorphisms and non-specific elevations in various inflammatory and metabolic conditions. Imaging modalities, including bone mineral density assessment, elastography of the liver and spleen, and the identification of Gaucher cells in bone marrow aspirates, provide supplementary evidence for establishing the diagnosis.

## 3 Therapeutic strategies and long-term management

The treatment of GD has shifted towards precise interventions based on disease classification, employing multimodal therapies to delay organ damage. The current treatment system centers around enzyme replacement therapy (ERT), combined with molecular chaperone therapy and individualized management.

### 3.1 Optimization and evidence-based application of ERT

ERT replenishes β-glucocerebrosidase exogenously, reduces the level of glucosylsphingosine, and ameliorates visceral and hematological abnormalities. Imiglucerase, a classic ERT drug, can increase hemoglobin by 30%–50% and reduce the volume of the liver and spleen (4). Velaglucerase alfa, a new ERT drug, has high internalization efficiency and low immunogenicity, reducing the splenic volume by 49.5% within 9 months (3). Guidelines recommend that high-risk patients initiate treatment with a high dose of 60 U/kg every 2 weeks, which can be reduced to 30–40 U/kg during the stable phase (21).

### 3.2 Progress in molecular chaperone therapy

Ambroxol, a small molecular chaperone drug, enhances the residual enzyme activity by stabilizing the conformation of the mutated β-glucocerebrosidase (GCase). It is suitable for patients with mild symptoms or those contraindicated for ERT. Studies have shown (22) that long-term treatment (0.5–6.5 years) can improve the symptoms of most patients, such as reducing the frequency of epistaxis and enhancing physical strength, while also increasing the levels of white blood cells and hemoglobin. However, the improvement of platelets depends on a significant reduction in splenic volume. Other studies have indicated (23) that ambroxol has varying efficacies for different mutation types. For example, it has a better effect on patients with missense mutations (such as N370S) and a limited effect on patients with nonsense mutations (such as c.1259G > A).

In this case, the compound heterozygous mutations (missense mutation in exon 7, c.907C > A, and nonsense mutation in exon 9, c.1259G > A) were treated with ambroxol, which improved the enzyme function. This outcome indicates the potential of targeted molecular chaperone therapy. In the future, it will be necessary to leverage multi-omics technologies to decipher the associations between genotypes and treatment responses, thereby optimizing individualized intervention strategies.

### 3.3 Exploration of emerging therapies

Eliglustat, a substrate reduction therapy drug, has been approved for adult patients with Type I Gaucher disease and can significantly reduce the level of Lyso-GL1 (a 72% decrease) (21), but data for children are insufficient. Adeno-associated virus (AAV)-mediated gene therapy has entered clinical trials (NCT052XXXX), and its safety has been preliminarily confirmed (24, 25). Hematopoietic stem cell transplantation (HSCT) is only applicable to specific infant patients, with a low 5-year survival rate (less than 40%) and a high treatment-related mortality rate (up to 25%) (26).

### 3.4 Research on long-term management protocols

Based on the Chinese Expert Consensus on Gaucher Disease in Children in 2021 and the updated international guidelines in 2023, research on long-term management protocols for Gaucher disease should focus on: (1) Multidisciplinary monitoring: Assess the volume of the liver and spleen (by ultrasound or magnetic resonance imaging), bone mineral density [by dual-energy x-ray absorptiometry (DXA)], and biomarkers (Lyso-GL1 and chitotriosidase) every 3–6 months. (2) Prevention and control of complications: For patients at high risk of bone diseases, supplement calcium (1,000 mg/d) and vitamin D3 (2,000 IU/d), and regularly screen for pulmonary arterial hypertension and myelofibrosis. (3) Psychosocial support: Establish a regional rare disease collaboration network, integrate medical insurance policies (such as critical illness insurance), charitable assistance, and psychological counseling to reduce the treatment interruption rate (the current continuous ERT treatment rate in China is only 58.7%) (27).

## 4 Analysis of inspirations and social impacts of cases

### 4.1 Analyzing the root causes of diagnosis and treatment delays and the breakthrough significance of early diagnosis

Gaucher disease (GD), a rare lysosomal storage disorder, presents with non—specific symptoms such as hepatosplenomegaly, cytopenia, and bone pain, posing diagnostic challenges. Consequently, the misdiagnosis rate in primary healthcare settings is as high as 60%—80%. A case in point is the first confirmed GD case in Xinjiang's Kashi region, where it took two years for a definitive diagnosis to be reached, with the patient being misdiagnosed with “chronic liver disease” and “idiopathic thrombocytopenia” in the interim. This case clearly reveals the dual problems of insufficient awareness of rare diseases and inadequate diagnostic techniques in remote areas.

In clinical practice, the non—distinctive nature of GD often results in missed or inaccurate diagnoses. So, when clinicians encounter patients with unexplained hepatosplenomegaly, thrombocytopenia, varying degrees of anemia, and bone pain, they need to be more vigilant. Initiating bone marrow analysis, enzyme activity assays, and genetic testing without delay is essential for accurate diagnosis. Early diagnosis and treatment, along with enhancing public awareness and education about rare diseases, can greatly increase the likelihood of timely and precise diagnosis and treatment. This is crucial for improving the prognosis and quality of life for patients with GD and other rare disorders.

Research shows that there's a significant positive correlation between delays in GD diagnosis and disease progression. Specifically, patients with a diagnostic delay of over one year face a 3.2—fold increased risk of developing osteonecrosis (1). This particular case stresses the importance of integrating enzyme activity detection (where β—glucocerebrosidase activity is just 12% of the normal value) with gene sequencing that spotted the compound heterozygous mutation c.907C > A/c.1259G > A in the GBA gene. This integration underlines the need to advance molecular diagnostic technology into resource—limited areas and sets up a standardized diagnostic path for similar cases. For individuals presenting with unexplained splenomegaly and cytopenia, initial enzyme and gene detection should be carried out to reduce the diagnostic period, preferably to under three months.

### 4.2 Optimization of stratified diagnosis and treatment strategies and challenges of economic toxicity

In terms of diagnostic methodologies, this case achieved an efficient and accurate diagnosis through advanced genetic testing and imaging techniques, demonstrating the potential application of novel technologies in remote areas and offering valuable insights for other medical institutions. Furthermore, the implementation of multidisciplinary collaboration models during the diagnostic process—evidenced by the close cooperation among pediatrics, hematology, genetics, and imaging departments—has reinforced the significance of multidisciplinary teams in diagnosing complex diseases and provided valuable experience and inspiration for medical teams globally.

The treatment strategy for Gaucher disease has evolved significantly, moving beyond the singular focus on enzyme replacement therapy (ERT) to a more comprehensive approach that considers genotype-phenotype characteristics. In this case, a high-dose regimen of ambroxol (25 mg/kg/d) was used as a transitional scheme before ERT. After six months of treatment, there was a significant increase in hemoglobin levels (43.4%, *P* < 0.001) and a substantial reduction in spleen volume (30.3%), highlighting the potential of ambroxol as a cost-effective alternative, especially in mild cases or primary care settings. Chinese research has indicated an effective rate of 92.85% for ambroxol treatment (2).

However, the high annual average cost of ERT, exceeding 2 million yuan, poses a substantial burden for families in Xinjiang, where the per capita disposable income was 28,735 yuan in 2023. This financial strain has led to 78.5% of patients interrupting their treatment. Looking ahead, a combined treatment strategy involving “ERT + SRT + molecular chaperone” is essential. For patients with missense mutations, ambroxol, which is significantly more affordable (only 1/20 the cost of ERT), should be prioritized. In contrast, for severe cases or those with nonsense mutations, a combination of ERT and Eliglustat (substrate reduction therapy) is advisable to optimize both therapeutic efficacy and economic viability.

### 4.3 Requirements for systemic change from the perspective of social ecology

The challenges in the disease management of GD profoundly reflect the systemic contradictions within China's rare disease healthcare system. While technological innovations have enhanced diagnostic efficiency, the imbalance in resource allocation has exacerbated economic toxicity, creating a stark contrast between the two aspects.

Take the Kashgar region in Xinjiang as an example. The scarcity of local medical resources and the low level of disease awareness severely hinder the early diagnosis and treatment of Gaucher disease. Although the first diagnosed case in Xinjiang, through the establishment of a regional multidisciplinary team (MDT) diagnostic and treatment model, integrating resources from pediatrics, hematology, and genetics, has demonstrated the transformative value of standardized diagnostic pathways in regions with weak medical resources, there are still numerous challenges and limitations in implementing systemic reforms.

In terms of treatment accessibility, the exorbitant medical costs pose a significant obstacle between patients and effective treatment. The average annual cost of ERT is as high as 400% of a patient's family annual income, pushing 45.6% of families into poverty due to treatment (5), which fully exposes the imperfections of China's rare disease protection system. Even with the continuous advancement of medical insurance policies, the cross-provincial settlement mechanism for rare disease expenses has not been fully established, and there are significant differences in medical insurance reimbursement ratios among different regions. As a result, some patients find it difficult to afford out-of-pocket expenses, severely compromising treatment compliance.

Reforms at the technological intervention level also face challenges. Although the rapid detection based on Lys0-GL1 (with a sensitivity of 98.2% and a specificity of 99.5%) and AI-assisted imaging diagnosis (with a splenic volume measurement error of <3%) can significantly improve the identification efficiency of primary healthcare institutions, the promotion of these technologies requires substantial financial investment in equipment procurement, personnel training, and technical maintenance. Primary healthcare institutions generally suffer from shortages of professional and technical personnel and outdated equipment, making it difficult to meet the application requirements of these technologies in the short term. In addition, the implementation of genotype-driven individualized treatment strategies, such as the use of high-dose ambroxol combined with dynamic monitoring for mild patients, has cost-benefit advantages. However, the implementation effects of this scheme may vary in different regions due to differences in medical levels. For patients with severe conditions or carrying nonsense mutations, the efficacy of the combination of ERT and substrate reduction therapy (SR) is limited by factors such as drug accessibility, the complexity of the treatment regimen, and individual patient differences. Moreover, the long-term safety and effectiveness of the combination treatment still require verification by more clinical data.

From the perspective of social ecology, achieving the reform goal of the “technology-policy-society” three-dimensional linkage is fraught with difficulties. Establishing a cross-provincial settlement system for rare disease expenses requires coordinating medical insurance policies, information systems, and financial interests among different regions, involving the collaboration of multiple departments, and the institutional design and implementation are extremely challenging. The construction of regional referral networks and teleconsultation platforms not only requires well-developed communication infrastructure and professional technical support teams but also faces issues such as inconsistent data sharing standards and unclear division of responsibilities among different medical institutions. In terms of public health education, although new media platforms have facilitated popular science work, the knowledge of rare diseases is highly specialized, making it difficult for the general public to understand. In addition, in grassroots areas, problems such as limited information dissemination channels and low health awareness among residents lead to the failure to achieve the expected popular science effects.

While it is confirmed that optimizing the diagnosis and treatment pathway with precision medicine and reconstructing the protection system with health equity are core approaches to solving the management dilemmas of rare diseases, in regions with limited global resources, it is necessary to fully consider and overcome the numerous challenges mentioned above. Only in this way can we truly form a replicable and sustainable “Chinese paradigm”.

## Data Availability

The original contributions presented in the study are included in the article/Supplementary Material, further inquiries can be directed to the corresponding author/s.
